# SLAMICP Library: Accelerating Obstacle Detection in Mobile Robot Navigation via Outlier Monitoring following ICP Localization

**DOI:** 10.3390/s23156841

**Published:** 2023-08-01

**Authors:** Eduard Clotet, Jordi Palacín

**Affiliations:** Robotics Laboratory, Universitat de Lleida, Jaume II, 69, 25001 Lleida, Spain; jordi.palacin@udl.cat

**Keywords:** ICP library, Iterative Closest Point, SLAM, mobile robot navigation

## Abstract

The Iterative Closest Point (ICP) is a matching technique used to determine the transformation matrix that best minimizes the distance between two point clouds. Although mostly used for 2D and 3D surface reconstruction, this technique is also widely used for mobile robot self-localization by means of matching partial information provided by an onboard LIDAR scanner with a known map of the facility. Once the estimated position of the robot is obtained, the scans gathered by the LIDAR can be analyzed to locate possible obstacles obstructing the planned trajectory of the mobile robot. This work proposes to speed up the obstacle detection process by directly monitoring outliers (discrepant points between the LIDAR scans and the full map) spotted after ICP matching instead of spending time performing an isolated task to re-analyze the LIDAR scans to detect those discrepancies. In this work, a computationally optimized ICP implementation has been adapted to return the list of outliers along with other matching metrics, computed in an optimal way by taking advantage of the parameters already calculated in order to perform the ICP matching. The evaluation of this adapted ICP implementation in a real mobile robot application has shown that the time required to perform self-localization and obstacle detection has been reduced by 36.7% when obstacle detection is performed simultaneously with the ICP matching instead of implementing a redundant procedure for obstacle detection. The adapted ICP implementation is provided in the SLAMICP library.

## 1. Introduction

The most important capabilities expected of a mobile robot are the ability to locate its position, follow a planned path, and detect and avoid unexpected obstacles appearing in the vicinity of the robot. Odometry is a direct computation method that provides a periodic update of the location of a mobile robot [1] based on the information of the angular velocity of the wheels and a precise estimation of the inverse kinematic matrix of the mobile robot [2]. However, this estimation method is prone to errors due to the structural uncertainties originated during the manufacturing process, which can lead to systematic odometry errors [3,4]. One example of such parameters affecting the inverse kinematics is the effective radius of the wheels [5], which may change depending on the air pressure in the wheel chamber (in the case of using wheels with an air chamber), the hardness of the rubber that is in contact with the ground, and the relative weight distribution of the robot. The effects of such uncertainties and some methods to calibrate the Inverse kinematic matrix have been deeply analyzed by the scientific community [6,7,8,9,10,11,12]. When the kinematics are calibrated, the relative displacement of a mobile robot can be estimated by multiplying the current estimation of the angular velocity of its wheels by its inverse kinematic matrix and the elapsed time since the last estimation. This relative cumulated displacement updates the position and orientation of the mobile robot and defines its trajectory.

Unavoidable external factors such as traction loss (wheel slippage or skidding) or the accuracy of the encoders used to monitor the angular velocity of the wheels [13,14,15,16] can heavily undermine the accuracy of this relative odometry. Alternatively, vision-based [17,18] and light detection and ranging (LIDAR) [19,20] are alternative computational methods that can be used to provide a periodic estimation of the absolute position of a mobile robot when wheel-speed-based odometry becomes too unreliable. In general, LIDAR odometry requires matching the point cloud generated by onboard 2D or 3D LIDARs with a reference point cloud that describes the scenario being explored, a procedure that is also known as geometric registration [21]. LIDAR-based odometry methods can be divided into three categories: based on the Iterative Closest Point (ICP) algorithm [22,23,24,25,26,27,28] introduced by Chen et al. [21] and Besl et al. [29], based on the detection of features [30,31,32,33,34,35], and based on deep learning methods [36].

ICP-based methods are based on the direct matching of the point cloud provided by a LIDAR sensor with a reference point cloud [37]. This approach tries to find the transformation matrix [21,29] that best minimizes the distance between active points of the LIDAR point cloud and the reference point cloud. Usually, the LIDAR point cloud is provided by a 2D or 3D scanner located onboard the mobile robot [38], and the reference point cloud contains a 2D or 3D representation of the area being explored. The ICP algorithm iteratively estimates the transformation matrix that minimizes an error metric, which is usually the sum of squared differences between the coordinates of the point clouds to be matched. For example, Bhandari et al. [39] proposed using the ICP algorithm to track the position of an object with a known geometry in a six degrees of freedom (DOF) registration problem from point cloud measurements.

Feature-based methods are centered on the extraction of recognizable geometric features from point clouds such as line segments, planes, and features in order to efficiently determine the existing correspondences [30,32,34,35,40]. This approach has the advantage of speeding up the iterative matching process [41], as a large point cloud can be simplified into a small number of characteristic geometric features, representing a significant reduction in the amount of information that must be handled during the matching process. Zang et al. [30] proposed the first feature-based matching method for fast, accurate LIDAR odometry and mapping (LOAM) using real-time LIDAR data. Qin et al. [35] further speeded up LOAM by relying only on good features to perform the feature selection.

Deep learning methods use artificial neural networks to operate with large amounts of data [36]. Deep learning methods have been applied to perform feature matching [42,43] and to estimate odometry using the point cloud as input data [44,45,46,47,48]. For example, Jung et al. [49] proposed a deep learning-based LIDAR odometry estimation method using a long-term recurrent convolutional network (LRCN), using point clouds as the input data for the deep learning algorithm. This approach processes spatial and temporal information at once by using a convolutional neural network (CNN) and a long-short-term memory (LSTM) artificial neural network. Their proposal was suitable for accurately estimating the position of a six-DOF robot from the point cloud information. 

This work is based on the use of the ICP algorithm to find the best transformation matrix to minimize the distance between the point cloud provided by the 2D LIDAR of an autonomous mobile robot and a reference point cloud that contains a 2D map describing the layout of the floor on which the robot is navigating. As stated previously, in cases where the map of the area is not available, the previous point cloud gathered by the mobile robot can be used as a reference, allowing the determination of the position difference between the coordinates at which the robot took the first scan and its current position. Then, the relative transformation matrix found by the ICP matching algorithm (which includes rotation and translation transformations) can be used to combine both point clouds into a single one in order to create a partial map description of the area in which the robot is navigating. This procedure can be repeated until the entire navigable area has been scanned, allowing the creation of a complete map of the application scenario [50]. The combined capability of providing simultaneous localization and mapping (SLAM [51]) is a feature widely implemented in mobile robots [52,53].

In general, the point cloud gathered from a 2D or 3D LIDAR sensor depicts a small area around the robot, but the reference point cloud describing or mapping a complete scenario tends to be very large. In such conditions, the main known drawback of using the ICP algorithm is the time required to iteratively find the best match between the point cloud gathered from a LIDAR and the map [54]. The optimized implementation of ICP matching has been addressed using different approaches [37]. For example, voxelization is a technique that divides the points contained in point clouds into small groups (or voxels) according to the normal distribution of each sub-point cloud. Voxelization is a simple yet effective approach to speeding up the ICP algorithm [55,56], although the reduction in the number of points also reduces the accuracy of the ICP matching [56]. The use of hardware acceleration is another effective approach to significantly reducing the execution time required by the ICP algorithm to find a suitable matching transformation [57].

Finally, the scan information provided by the LIDARs embedded in mobile robots is usually analyzed to detect and localize obstacles in the trajectory of the robot and, if required, update the map or implement specific obstacle avoidance procedures [58,59,60]. The exhaustive detection of discrepancies between the current LIDAR scan and the reference map is usually performed after ICP matching, as it requires the application of rotation and translation to the LIDAR scan points in order to fit them into the coordinates of the map. However, a closer look into the ICP matching algorithm reveals that this iterative procedure computes several variables that are relevant in the process of detecting discrepancies with the reference map. Therefore, the step of detecting the discrepant points again after the ICP matching could be optimized by taking advantage of the analysis already conducted as part of the ICP algorithm execution. Unfortunately, most ICP implementations available [22,23,24,25,26,27,28] discard all the internal variables after finishing the iterative process. The main goal of this work is to fuse the tasks of obstacle detection and mobile robot self-localization as a way to reduce the computational time required to detect obstacles around the mobile robot. This goal can be achieved by reusing the variables already computed during the detection of outliers as part of the ICP matching process.

### New Contribution

This work proposes speeding up the obstacle detection process in mobile robot navigation by directly monitoring outliers spotted after Iterative Closest Point (ICP) matching. In this context, the outliers are the discrepant points found after matching the point clouds provided in a partial LIDAR scan gathered by the mobile robot and a full facility map. Monitoring the outliers spotted after the ICP matching avoids the need to re-analyze the LIDAR scans to detect obstacles (discrepancies) with the facility map in mobile robot navigation. As a result, the time required to perform self-localization and obstacle detection in a real mobile robot application can be significantly improved. This work also presents the ICP implementation developed to return the list of outliers along with other relevant matching parameters and metrics. This optimized ICP-based obstacle detection agent designed to retrieve the list of outliers while simultaneously performing localization and mapping is freely available as SLAMICP [61]. This new ICP implementation is based on the LIBrary for Iterative Closest Point fitting (LIBICP [62]), developed by Geiger et al. [63] to analyze the KITTI Dataset [64,65]. Both ICP libraries are available under the GNU public license [66] and include MATLAB wrappers for fast prototype implementation and evaluation.

The paper Is structured as follows. Section 2 describes the materials and methods. In Section 3, the new adaptations implemented in the SLAMICP [61] library are presented in a detailed manner. Section 4 presents the experimental results obtained in an application with a real autonomous mobile robot. Final remarks are given in Section 5.

## 2. Materials and Methods

The materials and methods used in this work are: the mobile robot used in the validation experiments, the area in which the validation experiments have been carried out, the vanilla ICP algorithm, the reference computationally optimized LIBICP [62] library implementing the ICP algorithm, the reference trajectory defined to implement the validation experiments, the reference obstacles used, and the performance metric used to assess the improvement in execution time of the procedure used to detect obstacles during mobile robot navigation.

### 2.1. Mobile Robot

**Figure 1:**
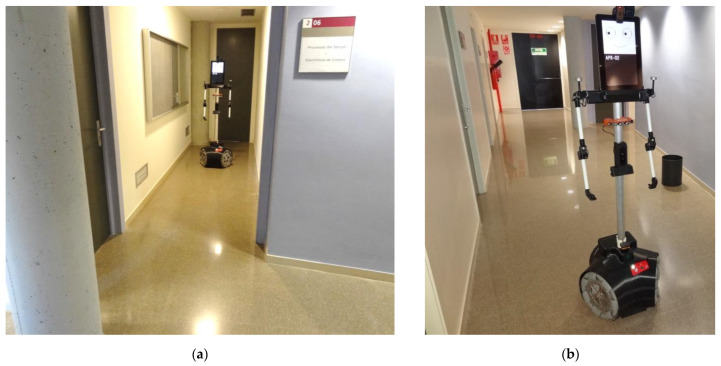
Two pictures of the mobile robot APR-02 while conducting the validation experiments: (**a**) starting position of the experiment; and (**b**) final position of the experiment.

The mobile robot used in this work is the APR-02, the second prototype of the APR robot series, developed by the Robotics Research Laboratory of the University of Lleida (Lleida, Spain) [67], which is shown in Figure 1. This mobile robot has been used to validate practical applications such as early gas leak detection [68] and enhance the sense of attention [69]. The robot has also been used as a platform test bench for alternative designs of omnidirectional wheels [70] and PID wheel control tuning [71]. The mobile robot embeds different sensors and actuators [67]: one 2D Hokuyo UTM-30 LIDAR for self-localization, one panoramic RGB camera on top of its head, three RGB-D Creative Senz3D cameras used for human interaction and ground validation, 8 fixed infrared distance detectors are used to provide interaction when the LIDAR is powered off; 16 passive infrared detectors are used to detect human activity and occupancy; 8 digital servomotors are used in the arms for gesticulation; redundant ambient sensors are used; and one touch screen is used to display an animated face and other interaction information.

### 2.2. Experimentation Area

The experimentation area in which the experiments have been carried out is located in the Polytechnic School of the University of Lleida, Spain. The validation experiments have been conducted at the end of the corridor on the second floor (see Figure 1a), which has a small lateral junction contiguous to the central corridor (see Figure 1b). This area was considered ideal for experimenting with obstacle detection because the intersection of the two corridors allows us to recreate a scenario where an obstacle suddenly appears near the path of the robot. Figure 1a shows the mobile robot ARP-02 located in a position that will be the starting point of a reference trajectory implemented in this work, and Figure 1b also shows the mobile robot in the middle of the corridor, near the ending position of the reference trajectory. Figure 1b also shows a wastebasket randomly placed in the corridor as an example of an obstacle appearing in the trajectory of the mobile robot.

### 2.3. Vanilla ICP Algorithm

The vanilla Iterative Closet Point (ICP) algorithm iteratively minimizes point-to-point distances between point clouds [29]. Given a point cloud M with K points describing a 2D or 3D map of the floor of a building.

(1) $$M=\left\{m_{1},\ldots ,m_{K}\right\},$$

and a partial point cloud T with L points describing partial spatial information, for example, obtained with a 2D LIDAR or 3D LIDAR:

(2) $$T=\left\{d_{1},\ldots ,d_{L}\right\}.$$

The ICP matching algorithm iteratively searches for the best match between the partial point cloud T and the map M of the scenario being explored. In each iteration, the ICP algorithm selects the closest points as correspondences and calculates the transformation $\left(R,t\right)$ that minimizes the following equation:

(3) $$E\left(R,t\right)=\sum_{i=1}^{K}\sum_{j=1}^{L}\omega_{i,j}{\left\|m_{i}-\left(Rd_{j}+t\right)\right\|}^{2},$$

where $\omega_{i,j}$ are the weights of a point-to-point match, which are assigned as $\omega_{i,j}=1$ if $m_{i}$ is the closest point to $d_{j}$ and $\omega_{i,j}=0$ if the distance between $m_{i}$ and $d_{j}$ is greater than a predetermined outlier threshold distance. Then, the outliers are the points $d_{j}$ in T that cannot be matched to any point in M.

**Figure 2:**
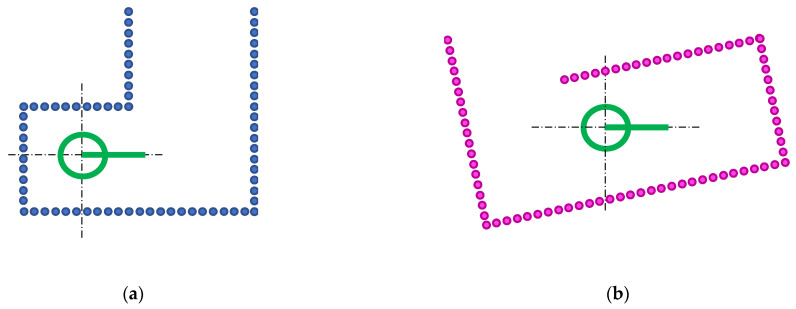
Schematic representation of the 2D point clouds gathered from a mobile robot (green circle): (**a**) reference partial point cloud M (blue dots) obtained at the starting position (0,0) and orientation 0°; and (**b**) partial point cloud T (magenta dots) obtained after the robot has moved.

Figure 2 shows the schematic representation of point clouds with no measurement errors, so the plot of the individual points that define the location of the walls looks like straight lines. Figure 2a represents an ideal reference or map point cloud M obtained by the 2D LIDAR of a mobile robot placed in a starting position. The assumption is that a complete map of the environment is not available. Figure 2b represents an ideal partial point cloud T obtained after the robot has moved. These 2D point clouds, M and T, can be expressed as:

(4) $$M=\left[\begin{array}{ccc} x_{1} & \ldots & x_{K}\\ y_{1} & \ldots & y_{K} \end{array}\right],$$

(5) $$T=\left[\begin{array}{ccc} x_{1} & \ldots & x_{L}\\ y_{1} & \ldots & y_{L} \end{array}\right].$$

The transformation $\left(R(\theta ),t\right)$ obtained by minimizing Equation (3) that defines the ICP matching represents the following information:

(6) $$(R(\theta ),t)=\left[\begin{array}{ccc} \cos\theta & -\sin\theta & t_{x}\\ \sin\theta & \cos\theta & t_{y}\\ 0 & 0 & 1 \end{array}\right];$$

where $t_{x}$ and $t_{y}$ are the estimates of the fitted (x,y) coordinates of the partial point cloud T in the map M, and the angle θ is the rotation that must be applied to the partial point cloud T to fit in the map M. The values $\left(t_{x},t_{y}\right)$ are relative to the (0,0) position defined in M, while the angle θ is relative to the initial orientation of the map; θ=0° means that no rotation is required in order to align T with M. The partial point cloud T transformed in order to fit in the coordinates of M is computed using:

(7) tT=cos ⁡θ−sin⁡θsin⁡θcos⁡θ·T+txty.

**Figure 3:**
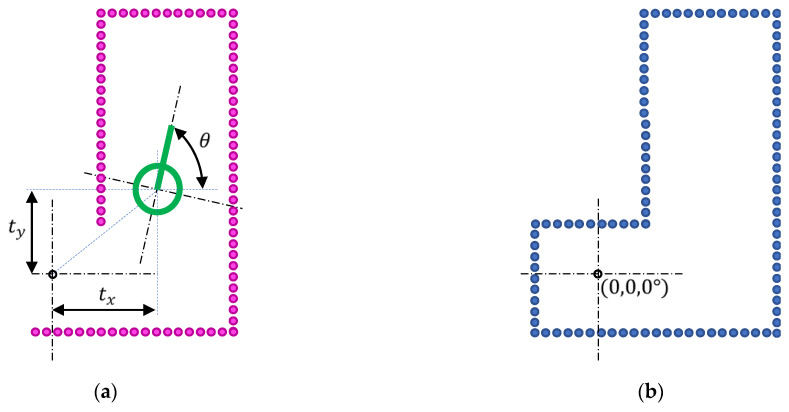
Interpretation of the results of the ICP matching: (**a**) representation of the transformed point cloud tT (magenta dots), where the position $\left(t_{x},t_{y},\theta \right)$ defines the self-localization of the mobile robot relative to the point (0,0,0°) of M; and (**b**) representation of the updated map uM (blue dots) created combining the transformed tT and M point clouds, to be used in the following ICP matchings.

Figure 3 shows the interpretation of the transformation obtained with ICP matching. Figure 3a shows the transformed point cloud tT obtained after applying the transformation $\left(R(\theta ),t\right)$ to the partial point cloud T. Figure 3a also shows the interpretation of the parameters of the transformation that define the self-localization of the mobile robot relative to the point cloud M. Finally, Figure 3b shows the result obtained when combining M (which only provides a partial description of the environment around the robot) and tT (which provides additional information about the environment around the robot). In this schematic example case, the result of the combination of these point clouds defines the complete map of the scenario uM that will be used as the reference map (M=uM) in the next ICP matching carried out later on. The specific procedure that allows the self-localization of the mobile robot (estimation of $\left(R(\theta ),t\right)$) and the creation of a detailed map of an application scenario (generate uM based on T) performs simultaneous localization and mapping (SLAM [51]), a procedure that is widely used in robotics requiring navigation from a known starting point to a destination and, for example, return to a charging station [72,73,74].

### 2.4. Reference LIBICP Library: Implementing the ICP Algorithm

The original ICP algorithm [29] implemented in the mobile robot APR-02 is based on the computationally optimized LIBICP [62] library proposed by Geiger et al. [63]. This ICP implementation iteratively minimizes point-to-point or point-to-plane distances between two point clouds; M and T. In mobile robotics, the point cloud T used to be the current 2D LIDAR scan gathered by the robot, and the point cloud M used to be the 2D map of the application scenario [68]. The LIBICP [62] library was created to evaluate the performance of stereo and optical flow systems in the definition of the KITTI Dataset [64]. 

The LIBICP [62] library is a cross-platform C++ library for fitting two 2D or 3D point clouds. This library takes advantage of a k-d tree search [75] implemented using the *array* and *multi-array* functions provided in the C++ Boost [76] library. These optimized functions require the minimum computational time to find the nearest neighbors between the points of two sets of point clouds. As a result of the matching, the LIBICP [62] library returns the transformation $\left(R(\theta ),t\right)$ that defines the best projection of the point cloud T over the point cloud M (minimizing Equation (3)). The LIBICP library does not return any performance metrics [77] or a list of inliers (points of the point cloud T that have matched correspondence in the map M) or outliers (points of T that do not have matching correspondence in the map M). As a consequence, other LIDAR scan procedures implemented in the APR-02 mobile robot, such as obstacle detection or map updating, require the development of redundant comparisons between the point clouds M and tT in order to detect, once again, the discrepant points, reducing the overall performance of the software agent implementing all these tasks.

### 2.5. Reference Trajectory Used in the Experimental Evaluation

**Figure 4:**
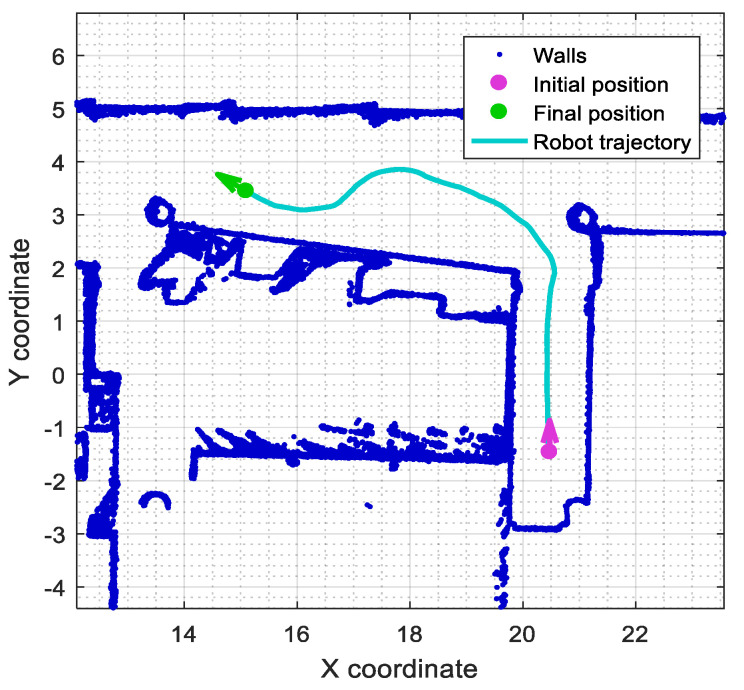
Reference trajectory of the mobile robot represented on the real 2D map of the experimentation area.

Figure 4 shows the path of the reference trajectory used to assess the obstacle detection performance obtained with the SLAMICP [61] library [78]. The trajectory is defined in a region of the 2D map depicting the experimentation area. In this case, the omnidirectional motion system of the APR-02 mobile robot is able to follow any trajectory defined in a 2D map by planning its path using fixed-distance spline interpolation [79]. 

The 2D point cloud map partially depicted in Figure 4 is composed of 10,402 points (blue dots) and was obtained by the mobile robot in a previous exploratory experiment [51]. The starting point (purple dot) defined in the reference trajectory is located near the end of a small, adjacent corridor (Figure 1a). From this position, the robot will perform a short straight displacement until reaching the main corridor. At this point, the robot will proceed to perform a set of soft and hard turns, describing an s-shaped path before reaching the final position (the green point) (see also Figure 1b). The performance of the ICP matching will be evaluated with the APR-02 mobile robot following this challenging reference trajectory in cases with and without stationary obstacles placed along the experimentation area.

### 2.6. Obstacle Definition

**Figure 5:**
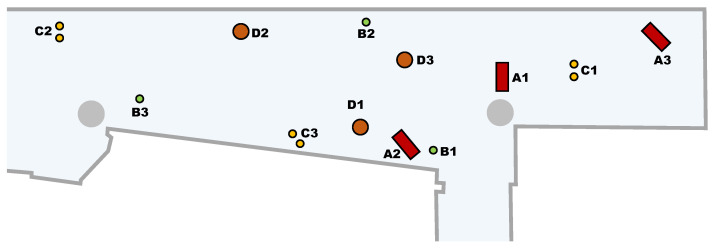
Depiction of the four different reference stationary obstacles (A, B, C, and D) used in this work. The number in the label identifies each one of the 3 different experiments conducted with an obstacle.

Figure 5 shows the approximate placement of the four stationary obstacles used in this work: A, a rectangular box of 480 × 215 mm; B, a small cylinder with a radius of 42 mm; C, two small cylinders, each with a radius of 50 mm; and D, a medium-sized cylinder with a radius of 135 mm. Each object has been placed in different locations along the corridor and labeled with a number. Each combination of obstacle and position will be used in the validation experiments. The obstacles have been placed in order not to block the reference trajectory of the robot in the different experiments.

### 2.7. Performance Metric: Computation Time

The metric used in this work to assess the improvement in execution time is based on monitoring the time taken by the software agent of the mobile robot to complete the following tasks: self-localization in the map (performing ICP matching), path-tracking, obstacle detection, and obstacle evaluation. 

The execution time improvement will be computed as:

(8) $$improvement=100-\left(\frac{t_{A-SLAMICP}*\;100}{t_{A-LIBICP}}\right),$$

where $t_{A-LIBICP}$ is the average computation time required by the software agent implemented in the mobile robot to process 2D LIDAR scans using the original LIBICP [62] library, and $t_{A-SLAMICP}$ is the average computational time required to process the same 2D LIDAR scans using the adapted SLAMICP [61] library developed in this work.

## 3. ICP Implementation: Returning the Outliers

This section details the new contributions implemented in the SLAMIPC [61] library relative to the reference LIBICP [62] library. The common inputs to both implementations are the 2D point cloud M used as a reference or map for the self-localization and the use of the current 2D LIDAR provided by the mobile robot as a partial point cloud T. The main differences between these two ICP implementations are the parameters returned. The scope of the new contributions will be highlighted by comparing the MATLAB wrappers provided to call the ICP functions of both libraries.

**Figure 6:**
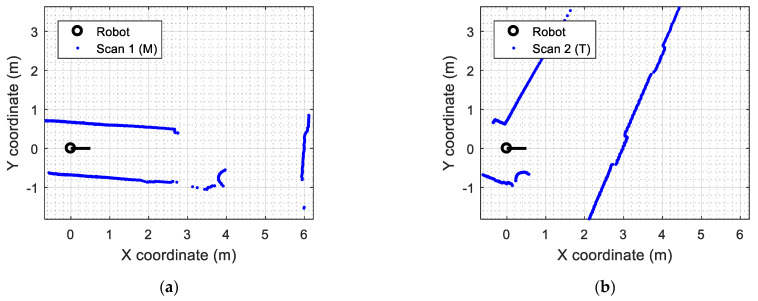
Real 2D scans gathered from the 2D LIDAR of the mobile robot: (**a**) the initial scan used as the reference point cloud M; and (**b**) scan T obtained after the mobile robot has moved.

Figure 6 shows two real scans, *M* and *T*, used in this section to illustrate the differences between the LIBICP [62] and SLAMIPC [61] libraries. Figure 6a shows a reference point cloud M obtained when the mobile robot is statically located and ready to start an exploration. Figure 6b shows the reference point cloud T obtained after the robot has moved. In both cases, these real 2D LIDAR scans are composed of 1081 raw points (blue dots) and represent the contour of the area around the mobile robot in two positions of the application scenario. Note that the scans displayed in Figure 6 are only composed of points, so they may seem noisy in some regions of the contour represented by a few dots. Additionally, the dots representing a plain wall are affected by the measurement error of the real 2D LIDAR, so these dots are not defining perfect flat surfaces.

### 3.1. Reference ICP Matching Library

The MATLAB wrapper that can be used to call the reference LIBICP [62] library is:

(9) $${Tr}_{fit}=icpMEX\left(M,\;T_{i},\;{Tr}_{estimated},indist,\;method\right),$$

where ${Tr}_{fit}$ is the transformation matrix (${Tr}_{fit}=(R(\theta ),t)$) required to match the i’th scan or partial point cloud $T_{i}$ with the reference point cloud M; ${Tr}_{estimated}$ is the transformation matrix that describes the last known position of the robot, which is used as an initial guess or approximation, usually this value is the ${Tr}_{fit}$ obtained during the previous matching; indist is a parameter that defines the distance threshold set to determine which points of T and M will be used as inliers during the matching process, its default value used in this work is 0.3 m; and method describes the matching strategy used by the ICP algorithm, which can be either point–point or point–plane.

**Figure 7:**
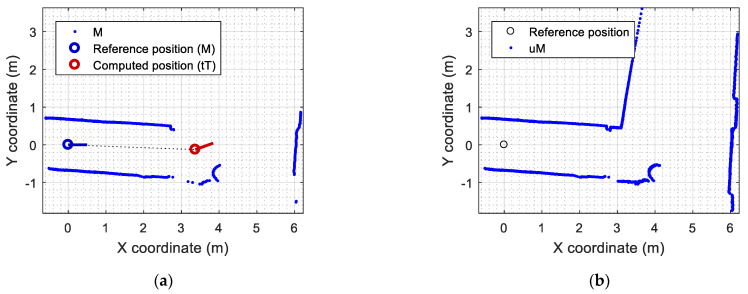
Results of the ICP matching using the LIBICP library: (**a**) representation of the self-localization of the mobile robot in the point cloud M; (**b**) update of the map uM based on the accumulation of tT, containing 2162 points.

Figure 7 shows the results obtained with the call to this wrapper function implementing ICP matching. Figure 7a shows the representation of the localization of the mobile robot relative to the point cloud M, deduced from the values of ${Tr}_{fit}$ (see Equation (6)). Additionally, Figure 7b shows the update of the map of the experimentation area M obtained by combining all the points of $T_{i}$ expressed in the coordinates of M (adding ${tT}_{i}$ to M with Equation (7)). This updated version of the map uM describing the experimentation area around the mobile robot used to have thick lines because of the accumulation of successive scans and the inaccuracy of the LIDAR measurements.

### 3.2. ICP Matching Improvement Returning the Outliers

The MATLAB wrapper that can be used to call the ICP matching procedure implemented in the new SLAMICP [61] library is:

(10) $$\left[P_{i},\;ni,\;md,\;O_{indx},O_{coords},\;tT,\;uM\right]=SLAMICP\left(M,\;T_{i},\;indist,\;method,iter,\;P_{i-1},\;Ot\right).$$

This wrapper function has the following new input parameters: iter is the maximum number of iterations given to the ICP algorithm to find the best solution; $P_{i-1}$ is the last known position and orientation of the robot defined as $\left(x_{i-1},y_{i-1},\theta_{i-1}\right)$; and Ot is an optional parameter that enables the definition of a specific distance threshold to detect the outliers after the ICP matching. This parameter is internally initiated to the value specified in the parameter indist if it is not specified in the call. This Ot parameter provides an additional degree of freedom to differentiate between inliers (points of $T_{i}$ that have been matched during the ICP procedure) and outliers (points of $T_{i}$ that have not been matched during the ICP procedure). In this work, the values used for indist and Ot are 0.3 m.

The new output parameters of the wrapper function are: $P_{i}$ is the direct estimate of the position and orientation of the robot $\left(x_{i},y_{i},\theta_{i}\right)$ in the map M; ni is the number of calls to the iterative fit function performed by the ICP algorithm, md is the mean distance of the inlier points after the ICP matching; $O_{indx}$ is a vector containing the indexed position of the points in $T_{i}$ that have been identified as outliers after the ICP matching, with values in a range from 1 to L; $O_{coords}$ is a 2D matrix containing the coordinates of the outliers detected in $T_{i}$ already transformed according to ${Tr}_{fit}$ so they can be plotted directly over the map M and/or added to M without requiring additional transformations; $tT_{i}$ is the matrix $T_{i}$ transformed according the matrix ${Tr}_{fit}$ so they can be plotted directly over the map M in order to highlight the location of the points of the current scan matched; and uM is an optional output value that, when requested, contains the concatenation of M and $O_{coords}$ in order to automatically generate an updated version of the map M. The output parameters $ni,\;md,\;O_{indx},O_{coords},\;tT,\;and\;uM$ are optional and only computed if requested.

The new SLAMICP [61] library also includes MATLAB wrappers with precompiled MEX files and application examples to use this wrapper in the Windows^®^ operating system. The development of the SLAMICP [61] has required the development of specific computer science work with the optimized C++ libraries used, as they were originally designed to return only one parameter as a result of the ICP matching.

**Figure 8:**
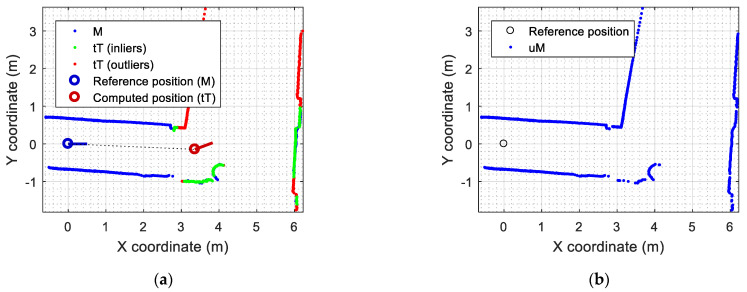
Results of the ICP matching using the SLAMICP library: (**a**) representation of the self-localization of the mobile robot in the point cloud M detailing the outliers (red points) and inliers (green points) of tT; (**b**) update of the map uM based on the combination of M and the outliers of tT, in this case containing 1617 points.

Figure 8 shows the application results that can be obtained with the SLAMICP [61] library. Figure 8a shows the representation of the self-localization of the mobile robot relative to the point cloud M, which is provided directly in $P_{i}$ as $\left(x_{i},y_{i},\theta_{i}\right)$ by the library, and the projection of the scan $T_{i}$ in the map M. Figure 8a details the points of the transformed scan ${tT}_{i}$ that have been identified as shared points or inliers (green points) and the points of the transformed scan ${tT}_{i}$ that have been identified as outliers (red points), describing new scenario information.

The detection of outliers after the ICP matching process can be used to optimize the map-building process. The outliers detected after the ICP matching depict new contour information that is not contained in the map. This means that only the outliers (new information) are merged with the current map. As an illustrative example, Figure 8b shows the result of updating the initial map of the experimentation area (Figure 8a, blue points) with the outliers detected in the current 2D LIDAR scan (Figure 8a, red points). This map-updating process rejects the inliers (Figure 8a, green points) in order to prevent the duplication of information in the map. Consequently, the point cloud map uM updated with only the outliers (Figure 8b) is composed of thin, clear contour lines. This is not the case when using all the points to compose the map, as any small discrepancy between M and tT will generate a cluster of noisy, redundant points in uM. Since the ICP algorithm is not capable of filtering noise while building the map, it is important that the map be created without dynamic obstacles.

### 3.3. Software Agent Implemented in the APR-02 Mobile Robot

This section describes the software agent implemented in the APR-02 mobile robot to perform self-localization, path-tracking, obstacle detection, and obstacle evaluation. This software agent is in charge of calling an ICP matching procedure with the current point cloud gathered by its onboard 2D LIDAR and the 2D map of the application scenario. Additionally, the APR-02 can be configured to create or update a 2D map M based on the information from the 2D LIDAR scans, although this procedure requires the definition of special exploratory missions focused on floor coverage [72,73].

**Figure 9:**
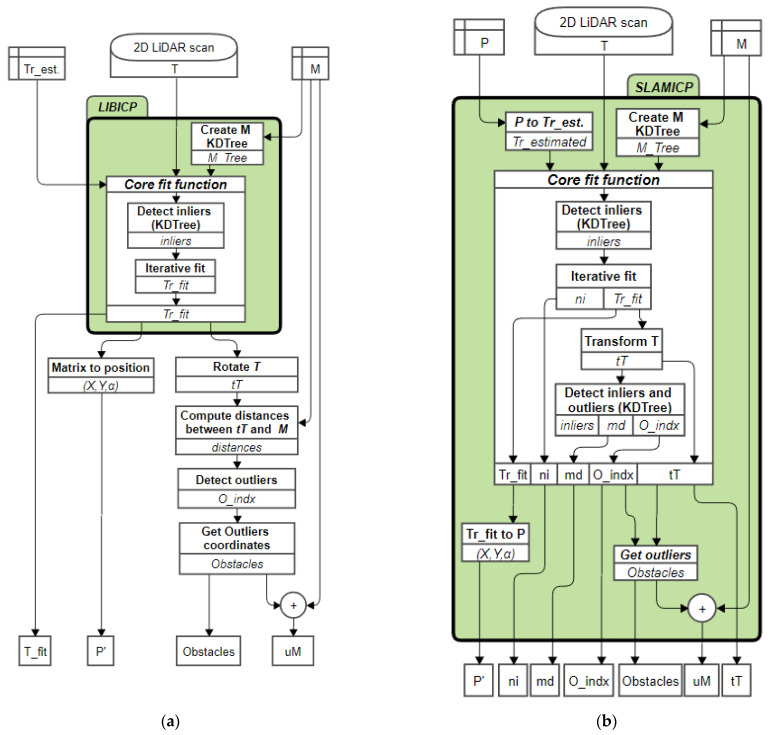
Flowchart of the software agent processing the 2D LIDAR scans of the APR-02 mobile robot: (**a**) original implementation based on the LIBICP [62] library; and (**b**) improved implementation using the adapted SLAMICP [61] library proposed in this work.

Figure 9a presents the original flowchart of the software agent implemented in the APR-02 mobile robot to process the point cloud information $T_{i}$ provided by the 2D LIDAR scanner. The set of instructions and functions included in the original LIBICP [62] library is outlined with a green, rounded frame. The disadvantage of this original implementation is that the localization of the obstacles in the 2D map requires performing a set of non-optimized subtasks, such as the comparison between the transformed T scan and the map M, to locate discrepancies. This subtask was especially inefficient, as the distance between M and tT was calculated by brute force instead of using an optimized k-d tree such as the one used internally in the LIBICP [62] library. However, the problem detected was that the LIBICP [62] library was not designed to return any other parameter than the transformation matrix of the matching.

A detailed analysis of the software agent presented in Figure 9a suggested the possibility of further improving its efficiency by using the k-d tree classification results. This improvement pointed us in the direction of adapting the LIBICP [62] library to reuse the internal classification of the points of T as inliers or outliers. This reuse was identified as an advantage that, for example, will also allow the computation of the distance between M and the transformed points of T without having to recalculate a new model tree.

Figure 9b shows the flowchart of the software agent based on the use of the new SLAMICP [61] library proposed in this work. The original and improved software agents perform the same subtasks and operations. As stated previously, the main differences are that the SLAMICP [61] library returns the list of outliers provided by the ICP matching algorithm and other matching parameters. This additional information prevents having to repeat time consuming tasks such as computing the distance between M and tT points with the sole purpose of detecting obstacles. Additionally, the SLAMICP [61] library operates in mobile robot coordinates P=(x,y,α) instead of using a transformation matrix ${tr}_{fit}$ and differentiates between outliers and inliers. The performance of the original and adapted software agents will be evaluated in Section 4.

The advantage of the SLAMICP [61] library is that it is fully oriented to mobile robot self-localization and SLAM, allowing the direct estimation of the position and orientation of the robot P=(x,y,θ) using the partial point cloud T and the map M as required inputs. Additionally, the SLAMICP [61] library dynamically determines which output parameters must be generated in order to keep computation time to a minimum. This is achieved by internally defining three different calls to the core ICP matching function.

The first call is designed to implement the basic computations. That is, the calculation of the transformation matrix (${tr}_{fit}$) that best minimizes the distance between M and active points of the template point cloud T, from which the new position of the robot P can be extracted. This call also retrieves the number of iterations ni performed by the ICP algorithm before finding the best ${Tr}_{fit}$, a metric that can provide useful information for analyzing the efficiency of the ICP algorithm. 

The second call includes the computation of the mean inliers distance (md). The inliers are the points of tT whose distance to the nearest point in M is lower than the specified threshold (indist). This operation requires transforming all the points in T according to ${Tr}_{fit}$ in order to obtain a transformed point cloud tT that can be plotted over the map M. At this point, the already implemented version of the k-d tree is used to determine the minimum distance between M and tT points. Finally, the mean inlier distance is obtained by dividing the accumulated distance by the number of active points detected in tT.

The third call is executed in the case of requesting the list detailing the index of the outliers ($O_{indx}$) detected in tT. In this case, the SLAMICP [61] library allows the definition of a threshold distance for outliers $O_{t}$ in order to differentiate them from the inliers. Additionally, this call also returns the coordinates of the outliers ($O_{coords}$). Therefore, there is no need to recalculate the projection of T in order to plot and visualize the outliers over the current 2D map M.

## 4. Results

This section presents the results of the different experiments carried out to assess the obstacle detection capabilities and execution time of the new SLAMICP [61] library. During this experimentation phase, the SLAMICP library is used only to identify the position of the robot and does not update or change the map of the area. The obstacles are analyzed based on the outliers (map discrepancies) between the current 2D LIDAR scan $T_{i}$ and the map M retrieved by the SLAMICP library. 

### 4.1. Obstacle Detection Performance at Different Translational Velocities

This section analyzes the number of outliers detected by the ICP matching procedure when the robot moves at a constant translational velocity. This experiment cannot be reproduced with other SLAM implementations, as only the SLAMICP [61] library returns the list of outliers detected. The outliers were detected matching the current 2D LIDAR scan, and a detailed 2D map of the area of experimentation describes the obstacles (or discrepancies) detected. The position of the outliers defines the position of stationary or mobile obstacles around the robot.

**Figure 10:**
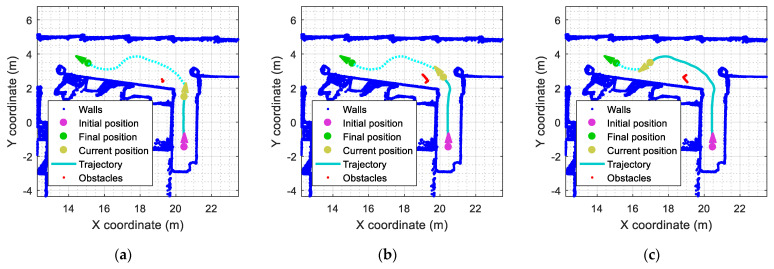
Representation of the outliers detected (red points) in the corridor (Figure 5, obstacle A2): (**a**) the robot is about to enter the corridor; (**b**) the mobile robot has entered in the corridor; (**c**) the robot has completed most of the planned trajectory.

As an example, the red dots presented in Figure 10 depict the outliers detected by the mobile robot while following the predefined path defined in Figure 4a. The obstacle used in this experiment is a rectangular box identified with label A2 in Figure 5. Figure 10 shows the evolution of the localization of the outliers detected. In Figure 10a, the mobile robot is about to enter the main corridor, and the obstacle A2 (a rectangular box located near the wall) is barely detected (red points). In Figure 10b, the mobile robot has entered the main corridor, and the outliers detected (red points) fully describe one side of the rectangular box. In Figure 10c, the mobile robot has almost completed the curved trajectory, and the outliers detected (red points) describe the other side of obstacle A2.

**Figure 11:**
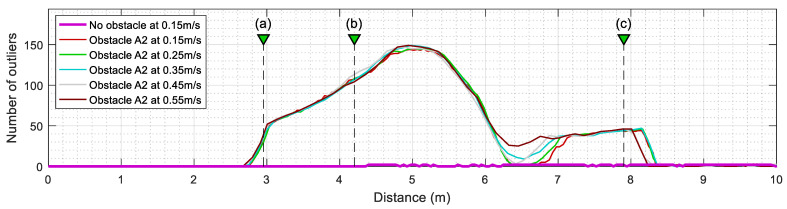
Evolution of the number of outliers (discrepant points) detected in the LIDAR scans after ICP matching when the mobile robot follows the reference trajectory at different velocities. The labels (a), (b), and (c) identify the positions of the robot depicted in Figure 10. Cases without obstacles (thick magenta line) and with the obstacle A2 (thin lines).

Figure 11 summarizes the evolution of the number of outliers detected by the ICP algorithm when the mobile robot is configured to repeat the same experiment at different translational velocities. This value is computed from the number of elements provided in $O_{indx}$, which contains the index of outliers identified by the SLAMIPC [61] library (see Equation (10)). The magenta line in Figure 11 shows the evolution of the outliers when no obstacle is located in the experimentation area. The movement of the robot, along with the inherent measurement errors of the LIDAR sensor, causes the false detection of a reduced number of outliers. The other lines show the evolution of the number of outliers detected when a rectangular box is in the corridor while the robot follows the reference trajectory at different translational velocities, ranging from 0.15 m/s to 0.55 m/s. To simplify the comparison between the different experiments, the y-axis of Figure 11 is set to indicate the distance traveled by the robot, a metric that is independent from the translational velocity of the robot and allows a direct visual comparison of the obtained results. Figure 11 shows that the translational velocity of the mobile robot does not have any remarkable impact on the number of outliers detected during the displacements.

**Figure 12:**
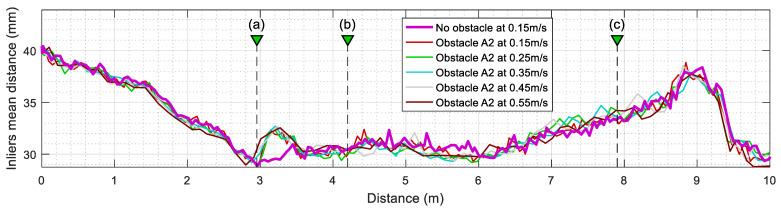
Evolution of the mean inliers (matched points) distance computed after ICP matching when the mobile robot follows the reference trajectory at different velocities. The labels (a), (b), and (c) identify the position of the robot depicted in Figure 10. Cases without obstacles (thick magenta line) and with the obstacle A2 (thin lines).

In a complementary way, Figure 12 summarizes the evolution of the mean inlier distance of active tT points after executing the ICP algorithm. This value is directly the output parameter md returned by the SLAMIPC [61] library (see Equation (10)). Figure 12 shows that the evolution of this mean inlier distance is virtually the same regardless of the translational velocity of the robot or the presence of obstacles. This is because the points that could worsen this parameter are classified as outliers and then excluded from the computation of this mean.

**Figure 13:**
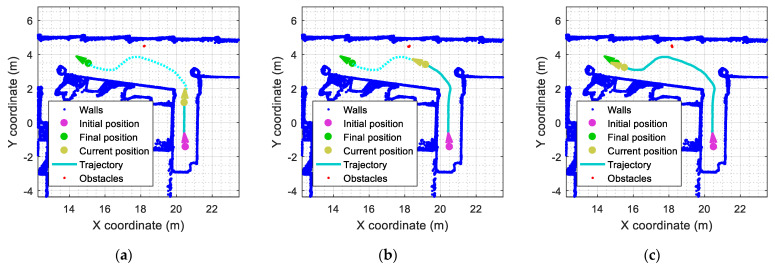
Detection of a small circular obstacle in the corridor (Figure 5, obstacle B2): (**a**) the robot is about to enter the corridor; (**b**) the mobile robot has entered the corridor; (**c**) the robot has completed most of the planned trajectory.

Figure 13 shows the evolution of the outliers registered in another example in which the mobile robot follows the reference trajectory (see Figure 4a). In this case, the obstacle used is the small cylinder identified with the label B2 depicted in Figure 5. In Figure 13a, the mobile robot is about to enter the main corridor, and the circular obstacle has already been detected (red points). In Figure 13b, the mobile robot has entered the corridor, and the front side of the circular obstacle is fully detected (red points). In Figure 13c, the mobile robot has completed most of the planned trajectory, and the outliers detected (red points) describe the back-side contour of the obstacle.

**Figure 14:**
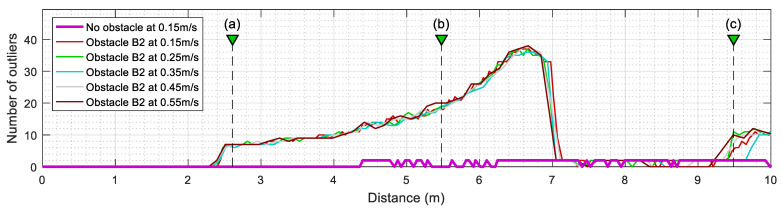
Evolution of the number of outliers (discrepant points) detected in the LIDAR scans analyzed. Results obtained at different translational velocities of the mobile robot. The labels (a), (b), and (c) identify the positions of the robot depicted in Figure 13. Cases without obstacles (thick magenta line) and with the obstacle B2.

**Figure 15:**
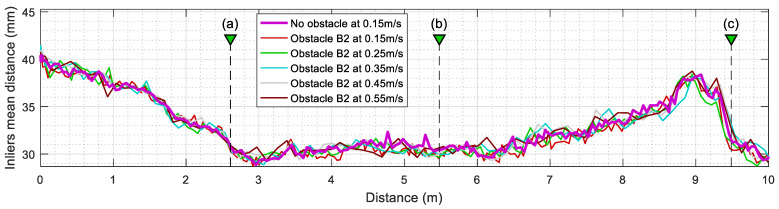
Evolution of the mean inliers (matched points) distance computed after ICP matching when the mobile robot follows the reference trajectory at different velocities. The labels (a), (b), and (c) identify the positions of the robot is depicted Figure 13. Cases without obstacles (thick magenta line) and with the obstacle B2 (thin lines).

Similarly, Figure 14 summarizes the evolution of the number of outliers detected by the ICP algorithm, and Figure 15 shows the evolution of the mean inlier distance when the mobile robot is configured to move at different translational velocities. The comparison of the evolution obtained with the obstacles A2 and B2 (Figure 11, Figure 12, Figure 14 and Figure 15) shows that these evolutions do not depend on the translational velocity of the mobile robot.

### 4.2. Obstacle Reconstruction

**Figure 16:**
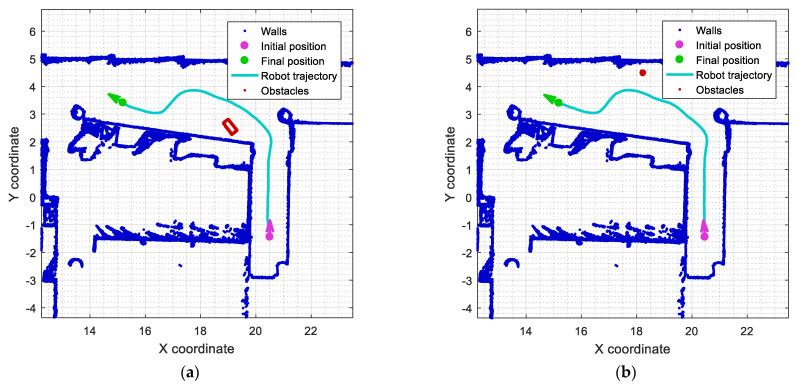
Examples of object reconstruction based on the cumulative projection of the outliers detected (red dots) during the motion of the robot: (**a**) rectangular obstacle A2 displayed in Figure 10; and (**b**) small circular obstacle B2 displayed in Figure 13.

This section describes the task of reconstructing the obstacles detected around the mobile robot. This reconstruction is based on the accumulation of outliers detected in each scan obtained by the robot during displacements. Figure 16 shows the reconstruction obtained by accumulating the outliers detected in two of the experiments conducted in the previous section. The reconstruction of the obstacles detected can be analyzed offline to determine whether or not the map of the building should be updated. In the case of small obstacles, the list of outliers detected must be filtered in order to provide a sharper reconstruction of the obstacles detected [58,80,81].

Figure 16a shows the cumulative projection of all the outliers detected during the experiment with the obstacle A2 described in Figure 10, depicting the shape of a rectangular box as an obstacle placed in the corridor. Similarly, Figure 16b shows the cumulative projection of all the outliers detected during the experiment with the obstacle B2 described in Figure 13, depicting the shape of a small circular object placed in the corridor and close to the walls. 

At this point, it must be noted that the layout of the floor is prone to change as furniture such as large flowerpots, chair benches, lounge chairs, water fountain machines, advertising stands, and others are often moved around. Then, from time to time, it may be necessary to update the reference map used by the mobile robot in order to accommodate such changes [82].

### 4.3. Improvement Evaluation

This section assesses the improvement in runtime achieved by the software agent implementing ICP matching in the APR-02 mobile robot. This evaluation compares the computational time taken by the original and proposed software agents to process the same 2D LIDAR scans (see Equation (8)). This evaluation is based on the offline analysis of the scans obtained by the mobile robot following the reference trajectory defined in Figure 4 in the cases including the stationary obstacles defined in Figure 5.

Table 1 shows the obstacles appearing in the trajectory of the mobile robot, the number of experiments performed with each obstacle, the maximum number of outliers detected, and the time taken by the software agent of the mobile robot to complete the following tasks: self-localization in the map (performing ICP matching), path-tracking, obstacle detection, and obstacle evaluation. Two of these experiments have been previously reported in Figure 10, with obstacle A2, and in Figure 13, with obstacle B2. In Table 1, $t_{A-LIBICP}$ is the average computation time required by the software agent to process one 2D LIDAR scan using the original LIBICP [62] library, and $t_{A-SLAMICP}$ is the average computational time required to process the same 2D LIDAR scans using the adapted SLAMICP [61] library developed in this work. In both cases, the ICP matching strategy is based on a point-to-line metric [21,29].

The analysis of average values in Table 1 shows that the number of outliers detected in the partial scan T does not significantly affect the computational time required by the software agent implemented in the APR-02 mobile robot to process the 2D LIDAR scans. The evaluation of the improvement shows that the use of the adapted SLAMICP [61] library has led to a significant reduction of 36.75% in the average computation time required to process a 2D LIDAR scan by the mobile robot. Basically, this improvement has been achieved by avoiding the need to repeat the calculation of discrepancies between the current scan and the map to detect obstacles around the mobile robot. This procedure is now based on the outliers returned by the SLAMICP [61] library that are processed as discrepancy points defining the localization of obstacles around the mobile robot.

## 5. Discussion and Conclusions

This work proposes speeding up the detection of obstacles in mobile robot navigation based on monitoring the outliers detected by the ICP matching procedure. The ICP matching algorithm [37] iteratively estimates the transformation matrix that best fits two related point clouds: the 2D LIDAR scan provided by a mobile robot and the 2D map describing the application scenario. The outliers are the points of the 2D LIDAR scan that cannot be matched with any point of the map due to being at a distance greater than a predetermined threshold distance.

In general, obtaining discrepancies between a 2D scan and a 2D map is a time-consuming process that requires the transformation of the 2D scan in order to fit it into the coordinates of the map, the analysis of the matching of the points, and the computation of the distance between points in order to determine which are located at a greater distance than a specified threshold. These time-consuming steps required to compute the variables needed to detect the map discrepancies are the same as the variables already computed during point cloud ICP matching. The SLAMICP [61] library presented in this work has been adapted to return the list of outliers while simultaneously performing localization and mapping. These outliers directly represent map discrepancies and can be monitored to detect obstacles and map changes around the mobile robot. Table 2 provides a comparison between the main features offered by the SLAMICP [61] library and other SLAM library packages such as Hector SLAM [83], Cartographer [84], and the reference LIBICP [62]. The main advantage of the SLAMICP [61] library is its implementation, which is tailored to return the list of outliers detected during ICP matching.

The performance of the software agent using the SLAMICP [61] library for mobile robot self-localization, path tracking, and obstacle detection has been evaluated by defining a reference trajectory composed of a combination of straight and curved displacements and a set of different obstacles. 

The results of the experimental evaluation conducted in this work have shown that the number of outliers detected while navigating is not affected by the translational velocity of the mobile robot. Another result obtained is that the mean inlier distance (the distance that defines the overall distance between the matched points) is not affected by the number of outliers because they are discarded and not included in the matching ($\omega_{i,j}=0$ discards the point $d_{j}$ of T). The appreciation of such effects is only possible using an ICP implementation that returns internal information detailing the evolution of the matching procedure. Additionally, the outliers returned after the ICP matching can be cumulated in order to reconstruct the shape of the obstacles detected around the robot, information that can be used to classify the obstacle and decide if the map should be updated. 

The final assessment of the APR-02 mobile robot has shown that the computational time required by the software agent processing the 2D LIDAR scans does not change significantly regardless of the number of outliers detected during the ICP matching. This is because the optimized k-d tree search [75] minimizes the computational time required to find the nearest neighbors between two sets of point clouds. Finally, the direct use of the SLAMICP [61] library to detect obstacles has led to a significant reduction of 36.7% in the average computation time required to process the 2D LIDAR scans. This is because monitoring the outliers reported by the ICP algorithm avoids the need to perform redundant analysis in order to detect obstacles (discrepant points between the current scan and the reference map of the application scenario). The implementation of the ICP library developed in this work is freely offered under the GNU General Public License [66] as the SLAMICP [61] library.

### Limitations and Future Work

The improvement achieved with the SLAMICP [61] library has been compared with the LIBICP [62] library, which was the ICP matching procedure originally used in the APR-02 mobile robot [85,86]. Future research will address the assessment of the computational performance of the SLAMICP [61] library under other specific layout conditions and hardware usage [87,88,89]. 

Future developments of the SLAMICP [61] library will address enhancing the performance of the ICP matching in the case of registering a 2D point cloud gathered from a push-broom or articulated 2D LIDAR [38] using a 3D map as a reference and will analyze the correlation between the evolution of the number of outliers detected and the situational awareness achieved in dynamic environments [90]. These developments may allow for improved interpretation of the ICP matching results in crowded and unstructured environments and in multistory navigation.

## Figures and Tables

**Table 1:** Mean time spent by the software agent of the APR-02 mobile robot using the LIBICP [62] and the SLAMICP [61] libraries depending on the obstacle appearing in the trajectory of the robot.

Obstacle	Trajectory Experiments	Maximum Number of Outliers Detected	LIBICP [62]: $t_{A-LIBICP}$ (ms)	This Work: $t_{A-SLAMICP}$ (ms)	Improvement
A1	3	82	71.06	45.33	36.21%
A2	3	45	69.29	43.71	36.92%
A3	3	24	70.48	44.43	36.96%
B1	3	41	70.52	44.58	36.78%
B2	3	29	68.78	43.39	36.91%
B3	3	43	70.49	44.64	36.67%
C1	3	23	71.36	44.66	37.42%
C2	3	12	70.56	44.41	37.06%
C3	3	44	70.52	44.45	36.97%
D1	3	63	70.33	45.01	36.00%
D2	3	44	70.51	44.74	36.55%
D3	3	123	70.35	44.61	36.59%

**Table 2:** Comparison of the features of the SLAMICP [61] library.

Algorithm	Linux/ROS	Windows/Matlab	Map Data-Type	Automatic Map Update	Retrieves the List of Outliers
LIBICP [62]	Yes	Yes	Point cloud	No	No
SLAMICP [61]	Yes	Yes	Point cloud	Yes	Yes
Hector SLAM [83]	Yes	No	Grid	Yes	No
Cartographer [84]	Yes	No	Grid	Yes	No

## Data Availability

The data used in this work and the SLAMICP library are available at [61]: http://robotica.udl.cat/slamicp/ (accessed on 31 July 2023).
